# Differential gene expression and miRNA regulatory network in coronary slow flow

**DOI:** 10.1038/s41598-024-58745-w

**Published:** 2024-04-10

**Authors:** Lihua Sun, Juan Wang, Jimin Lei, Ying Zhang, Yue Zhang, Yaling Zhang, Shifeng Xing

**Affiliations:** 1 Department of Cardiology, Zhongshan Boai Hospital Affiliated to South Medical University, No. 6, Chenggui Road, Zhongshan, 528405 Guangdong China; 2 Department of Cardiology, The Fifth Affiliated Hospital of Xinjiang Medical University, No. 118 Henan West Road, Xinshi District, Urumqi, 830000 Xinjiang China

**Keywords:** Coronary slow flow, Cell proliferation, FPR1, miR-342-3p, Cardiology, Diseases

## Abstract

Coronary slow flow (CSF) is characterized by slow progression of coronary angiography without epicardial stenosis. The aim of this study was to explore the potential biomarkers and regulatory mechanism for CSF. Peripheral blood mononuclear cells from 3 cases of CSF and 3 healthy controls were collected for high-throughput sequencing of mRNA and miRNA, respectively. The differentially expressed mRNAs (DE-mRNAs) and miRNAs (DE-miRNAs) was identified. A total of 117 DE-mRNAs and 32 DE-miRNAs were obtained and they were mainly enriched in immune and inflammatory responses. Twenty-six DE-mRNAs were the predicted target genes for miRNAs by RAID, and then the regulatory network of 15 miRNAs were constructed. In addition, through the PPI network, we identified the three genes (FPR1, FPR2 and CXCR4) with larger degrees as hub genes. Among them, FPR1 was regulated by hsa-miR-342-3p, hsa-let-7c-5p and hsa-miR-197-3p and participated in the immune response. Finally, we validated the differential expression of hub genes and key miRNAs between 20 CSF and 20 control. Moreover, we found that miR-342-3p has a targeted regulatory relationship with FPR1, and their expression is negatively correlated. Then we established a hypoxia/reoxygenation (H/R) HUVEC model and detected FPR1, cell proliferation and apoptosis. Transfection with miR-342-3p mimics can significantly promote the proliferation of HUVEC under H/R conditions. FPR1 were associated with CSF as a biomarker and may be regulated by miR-342-3p potential biomarkers.

## Introduction

Coronary slow flow (CSF) is a special phenomenon of coronary microangiopathy, which is characterized by normal or near normal coronary angiography and slow progression of contrast agent into distal vessels^1^. The incidence of CSF was reported to be 7% in patients with various suspected cardiovascular diseases for coronary angiography^2^. The total cholesterol, low density lipoprotein C level, body mass index, incidence of metabolic syndrome and hospitalization rate of CSF patients were significantly higher than those of the control group^3^. Slow blood flow is often associated with adverse cardiovascular events, including sudden cardiac death, indicating a poor prognosis^4,5^. Therefore, early diagnosis and prevention of CSF are valuable.

The pathogenesis of CSF remains unclear. A large number of studies have shown that the pathogenesis of CSF may be related to vascular endothelial dysfunction, thrombosis, obesity, atherosclerosis and other factors^6,7^. Recent studies have revealed the role of inflammation, and it is currently believed that CSF is the result of coronary microvascular changes caused by chronic inflammation^8,9^. Serum uric acid level, hypertension, low HDL-c level and high hemoglobin level have been reported to be associated with the onset of CSF^7,10^. Due to limited understanding of the pathogenesis of CSF, there is currently no definite treatment for CSF, and there is a lack of effective biomarkers to specifically predict CSF.

In recent years, high-throughput sequencing technology has been widely used in the study of cardiovascular diseases^11,12^. High-throughput sequencing technology will help further elucidate the molecular mechanisms of cardiovascular diseases, which may lead to safer and more effective treatments^13^. In addition, microRNA (miR) is becoming a key regulator of phenotypic changes associated with physiological and pathological background in various cardiovascular diseases^14^. To our knowledge, few studies have explored the molecular regulation mechanism of CSF and biomarkers through gene transcriptome combined with miRNA sequencing.

Therefore, the focus of this study is to investigate the molecular regulation mechanism and potential biomarkers of CSF through high-throughput sequencing analysis of mRNAs and miRNAs.

## Materials and methods

### Sample collection

Peripheral blood samples from 43 patients with CSF and 43 healthy controls hospitalized in 2018 were collected from the Fifth Affiliated Hospital of Xinjiang Medical University. CSF patients were screened with reference to the evaluation of the flowmeter frame method according to Gibson et al.^15^ in the coronary artery angiography (CAG) examination. CSF was diagnosed by at least one coronary flow frame count greater than 27 frames/s at 30 frames/s recording speed. Exclusion criteria: (1) Patients with severe luminal coronary artery stenosis or other coronary artery diseases, such as coronary artery aneurysm, coronary spasm, and calcification; (2) The patient who was diagnosed with coronary artery disease after CAG and underwent percutaneous coronary intervention; (3) Patients with myocardial infarction, hypertrophic cardiomyopathy, restrictive cardiomyopathy, congenital heart disease, dilated cardiomyopathy; (4) Patients with hypothyroidism and/or hyperthyroidism, liver and kidney failure, chronic obstructive pulmonary disease, malignancies, autoimmune diseases, and acute or chronic infectious diseases. Another randomly selected healthy people who participated in the physical examination during the same period and underwent CAG examination, showing completely normal coronary arteries and normal blood flow, were considered as the control group. All subjects read and signed the informed consent form. The study was in conformance with the guidelines of the 1975 Declaration of Helsinki, and was approved by the ethics committee of the Fifth Affiliated Hospital of Xinjiang Medical University.

### RNA extraction

The peripheral blood samples of three CSF patients and three healthy controls were randomly selected for follow-up experiments. Peripheral blood samples were treated with separation solution (TBD, Tianjin, China) to collect peripheral blood lymphocytes. Trizol (Invitrogen, California, USA) was used to extract total RNA from lymphocytes. The purity of RNA was detected by OD260/280 ratio, and the quality of RNA was detected by gel electrophoresis.

### MiRNA sequencing and data analysis

After RNAs qualification, sequencing libraries were constructed using Multiplex Small RNA Library Prep Set for Illumina (NEB, USA) following manufacturer’s recommendations. Briefly, 3ʹ SR adaptor was directly, and specifically ligated to 3ʹ end of miRNA, siRNA and piRNA. Then first strand cDNA was synthesized using M-MuLV Reverse Transcriptase (Promega, Madison, USA). The library preparations were sequenced on an Illumina Hiseq 2500/2000 platform and 50 bp single-end reads were generated. After quality control of raw data, miRNA expression levels were counted and normalized by transcript per million (TPM).

### RNA sequencing and data analysis

Sequencing libraries were generated using NEBNext® UltraTM RNA Library Prep Kit for Illumina (NEB, USA) following manufacturer’s recommendations. The first strand cDNA was synthesized using M-MuLV Reverse Transcriptase (Promega, Madison, USA). Second strand cDNA synthesis was subsequently performed using DNA Polymerase I and RNase H. The library preparations were sequenced on an Illumina Novaseq platform and 150 bp paired-end reads were generated. After quality control of raw data, featureCounts was used to count the reads numbers mapped to each gene. We quantitatively analyzed the gene expression level of each sample through calculating the FPKM, and then combined to obtain the expression matrix of all samples.

### Differentially expressed genes

Differential expression analysis for miRNA and mRNA was performed using the DESeq2 R package. RNAs with an |log2(FoldChange)| > 1 and P-value < 0.05 were assigned as differentially expressed miRNAs (DE-miRNAs) between CSF and control. The |log2(FoldChange)| > 0 and adjusted P-value < 0.05 were identified as differentially expressed mRNAs (DE-mRNAs) between CSF and control.

### Enrichment analysis

Gene Ontology (GO) and Kyoto Encyclopedia of Genes and Genomes (KEGG)^16^ enrichment analysis of DE-mRNAs were implemented by the ClusterProfiler R package. GO terms included biological processes (BP), cellular composition (CC) and molecular function (MF). In addition, for the gene set enrichment analysis (GSEA) all genes were incorporated into the analysis using the ClusterProfiler R package. The adjusted P-value < 0.05 were considered significantly enriched.

### Construction of protein–protein interaction (PPI) network

The differentially expressed mRNAs were put into STRING (v12.0, https://STRING-db.org) online tool, and the combined score > 0.4 was considered significant. The PPI network was visualized by Gephi software (v0.10, https://gephi.org/). The hub genes were chosen based on their degree of connectivity with other genes.

### Target prediction and drug prediction

The mRNA-miRNA interaction pairs with setting score ≥ 0.5 were collected from RAID v2.0 database (www.rna-society.org/raid/)^17^. Then target mRNAs of DE-miRNAs was obtained from these interaction pairs. The targeted drugs of hub genes were predicted using the therapeutic target database (TTD, https://db.idrblab.net)^18^ to facilitate drug discovery.

### Cell culture and treatment

Human umbilical vein endothelial cells (HUVEC; iCELL, Shanghai, China) were cultured in DMEM with 10% fetal bovine serum (FBS), and 1% penicillin–streptomycin at 37 °C in a 5% CO_2_. To establish a hypoxia/reoxygenation (H/R) model, Cell culture medium was replaced with serum-free DMEM and incubate it in a hypoxic environment of 95% N_2_-5% CO_2_ for 24 h. Remove the cells from the hypoxic environment, replace the culture medium with DMEM supplemented with 10% FBS, and perform reoxygenation culture in an environment of 95% air − 5% CO_2_ for 6 h. HUVEC was transfected with miR-342-3p mimics, miR-342-3p inhibitor, and a corresponding negative control (NC) RNA using the Lipofectamine 2000 Reagent (Invitrogen).

### Dual-luciferase assay

The binding site of FPR1 for hsa-miR-342-3p and hsa-miR-197-3p were obtained from RAID v2.0 database. The 3′ UTR binding sequence of wild-type (FPR1-WT) and mutant sequence (FPR1-MUT) were cloned into the luciferase vector pmirGLO vector (Promega, Madison, USA), respectively. Then, 293T cells were cotransfected with the pmirGLO vector and miRNA mimics (GenePharma, Shanghai, China) using the Lipofectamine 2000 Reagent (Invitrogen). Cell lysates were collected after 24 h and the luciferase activity was determined using Dual-Luciferase Reporter System (Promega).

### Cell proliferation and apoptosis

For cell proliferation detection, HUVEC in each group were incubated with 10 μM BrdU for 24 h and then fixed with 4% paraformaldehyde for 10 min. Then, the staining reagent from the EdU kit (Beyotime, Shanghai, China) was used follow the manufacturer’s instructions to stain the EdU labeled cells. DAPI was used to counterstained the nucleus. A fluorescence microscope (OLYMPUS, IX71) was used to examine the cells.

In addition, cell proliferation was assessed using the Cell Counting Kit-8 (CCK-8, Beyotime) according to the manufacturer’s instructions. Briefly, for HUVEC in each group, CCK-8 solution (10 µl) was added to each well containing 100 µl of culture medium, followed by incubation at 37 °C for 2 h. The absorbance at 450 nm was measured using a microplate reader to determine cell viability.

For cell apoptosis detection, HUVEC were fixed with 4% paraformaldehyde for 10 min and infiltrated with 0.1% Triton X-100 in PBS. Cells were covered with TUNEL reaction solution (Solarbio, Beijing, China) at 37 °C for 1 h in dark. Afterwards cells were washed with PBS, it will counterstain using DAPI. Images were generated using fluorescence microscope (OLYMPUS, IX71).

### Quantitative real-time polymerase chain reaction (qRT-PCR)

The total RNA was isolated from 86 lymphocytes samples (43 CSF and 43 control). Reverse transcription for mRNA and miRNA expression was performed using PrimeScript™ RT Master Mix (TaKaRa, Dalian, China) and miScript Reverse Transcription Kit (Qiagen, Dusseldorf, Germany), respectively. The qRT-PCR was carried out using the SYBR Green Master Mix (Invitrogen) according to the manufacturer. The primer sequence of genes was shown in Table 1. Relative expression of mRNA and miRNA was calculated using 2^–ΔΔCT^ method. Genes were normalized to GAPDH and U6.

**Table 1:** The primer sequence for genes.

Genes	Primers
hsa-miR-197-3p	F: 5′-ACACTCCAGCTGGGTTCACCACCTTCTCCA-3′
	R: 5′-TCGTGGAGTCGGCAATTCAGTTGAGGCT-3′
Hsa-let-7c-5p	F: 5′-TGAGTAGTAGGTTGTGTT-3′
	R: 5′-GCTGTCAACGATACGCTACCTA-3′
hsa-miR-342-3p	F: 5’-TGCGGTCTCACACAGAAATCGCAC-3’
	R: 5’-CCAGTGCAGGGTCCGAGGT-3’
U6	F: 5′-CTCGCTTCGGCAGCACA-3′
	R: 5′-AACGCTTCACGAATTTGCGT-3′
FPR1	F: 5′-GCCCGTTCTTTACATTGCAT-3′
	R: 5′-CGTCCCGTAGACAAAATGGT-3′
FPR2	F: 5′-CCTTATAGTCTTGAGAGAGCCCTGA-3′
	R: 5′-TGCAGGAGGTGAAGTAGAACTGG-3′
CXCR4	F: 5′-CCACCATCTACTCCATCATCTTC-3′
	R: 5′-ACTTGTCCGTCATGCTTCTC-3′
GAPDH	F: 5′-TGTGGGCATCAATGGATTTGG-3′
	R: 5′-ACACCATGTATTCCGGGTCAAT-3′

### Western blot

Total protein was extracted radioimmunoprecipitation (RIPA) lysis buffer and quantified using BCA Protein Assay Kit (Beyotime). An equal amount of protein (30 μg) from each sample mixed with 5× SDS sample buffer and heated at 100 ℃ for 5 min, followed by SDS-PAGE electrophoresis. The proteins on the gel were transferred to the PVDF membrane by wet method. Membrane was incubated with 5% skim milk at room temperature for 1 h to prevent non-specific binding. The membrane incubated in TBST solution with primary antibody (ABclonal, Wuhan, China) at 4 ℃ for 12 h. Afterwards, the membrane was incubated in containing HRP-conjugated secondary antibody at room temperature for 2 h. ECL detection reagents was added to the membrane for testing protein bands through ChemiScope 5300 Pro. β-actin was used as an internal control to calculate the relative expression of FPR1 by ImageJ (v10.2, NIH, CA, USA).

### Statistical analysis

Data analysis was used SPSS v20.0 software. Data were presented as mean ± standard deviations (SD). Student *t*-test was used to compare the differences between CSF and control group. The P-value < 0.05 was considered statistically significant. Test level α = 0.05 (two-sided). Correlation analysis was performed using the Pearson correlation method.

### Ethical approval

The study was approved by the ethics committee of the Fifth Affiliated Hospital of Xinjiang Medical University (XYDWFYLSk-2020-012). Written informed consent was obtained from each participant before authors commenced any samples collection.

## Results

### Clinical features of samples

**Figure 1:**
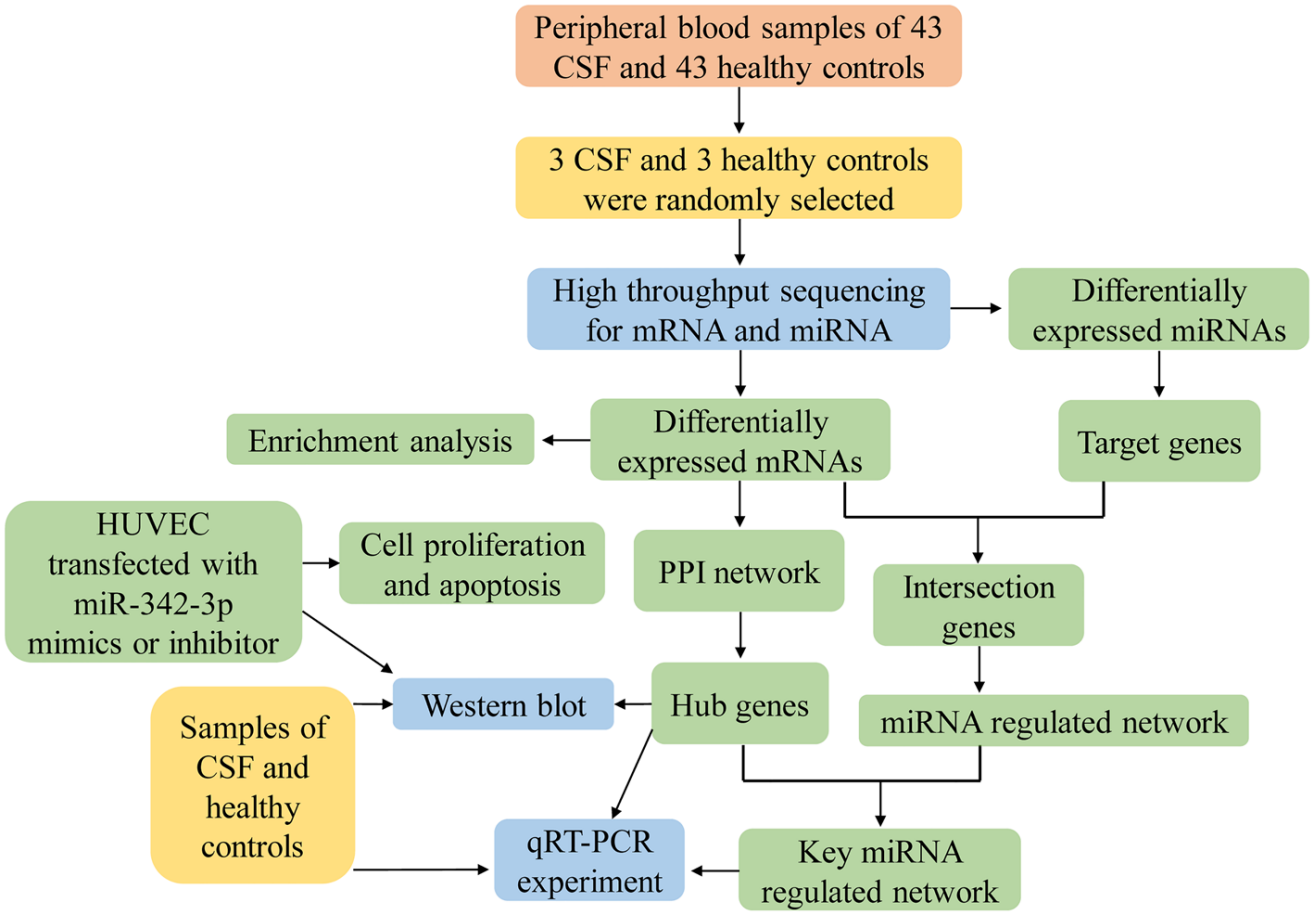
The flowchart of this study. *CSF* coronary slow flow, *PPI* protein–protein interaction, *qRT-PCR* quantitative real-time polymerase chain reaction.

The graphical abstract is shown in Fig. 1. A total of 6 peripheral blood lymphocytes samples, including 3 CSF and 3 normal control samples were collected and analyzed by Illumina sequencing for mRNAs and miRNAs. The clinical features of all samples were analyzed (Table 2).

**Table 2:** The clinical features of CSF patients and healthy group.

Variables	CSF (n = 43)	Control (n = 43)	t/χ^2^	*P*
Age	54.38 ± 8.86	57.24 ± 8.35	0.36	0.69
History of diabetes mellitus (n, %)	10 (23.25)	6 (13.95)	0.14	0.58
History of hypertension (n, %)	27 (62.79)	26 (60.47)	0.37	0.85
Smoking history (n, %)	15 (34.88)	13 (30.23)	0.2118	0.6453
Abnormal FBG (n, %)	14 (32.56)	7 (16.28)	3.087	0.0789
Dyslipidemia (n, %)	17 (39.53)	9 (20.93)	3.528	0.0603
BMI (kg/m^2^)	25.08 ± 2.07	24.82 ± 2.37	0.5418	0.5894
FBG (mmol/l)	5.67 ± 1.02	5.44 ± 1.52	2.05	0.11
2hPG (P25–P75 mmol/l)	5.62 ~ 9.18	5.69 ~ 7.83	0.80	0.85
HbA1c (mmol/l)	6.48 ± 0.72	6.07 ± 0.77	2.550	0.0126
WBC (× 10^9^/l)	6.84 ± 1.18	6.29 ± 1.03	2.303	0.0238
PLT (× 10^9^ l)	203.28 ± 36.73	227.48 ± 41.68	2.856	0.0054
TC (mmol/l)	4.21 ± 0.94	4.25 ± 0.82	0.28	0.84
TG (mmol/l)	1.48 ± 0.82	1.46 ± 0.73	1.56	0.28
LDL-C (mmol/l)	2.73 ± 0.64	2.64 ± 0.61	2.32	0.15
HDL-C (mmol/l)	1.18 ± 0.42	1.09 ± 0.33	1.46	0.43
Apo-A (g/l)	1.14 ± 0.22	1.07 ± 0.21	1.29	0.37
Apo-B (g/l)	0.91 ± 0.18	0.93 ± 0.09	2.61	0.11
URIC (umol/l)	349.12 ± 63.67	344.28 ± 83.29	1.46	0.52
BUN (mmol/l)	5.38 ± 1.21	5.29 ± 1.37	2.27	0.08
Cr (umol/l)	75.40 ± 10.25	79.28 ± 11.42	1.83	0.21

The values are shown as mean ± standard deviation.

*FBG* fasting blood glucose, *BMI* body mass index, *2hPG* 2 h postprandial blood glucose, *HbA1c* glycated hemoglobin, *WBC* white blood cell count in blood routine, *PLT* platelet count, *TC* total cholesterol, *TG* triglyceride, *LDL-C* low density lipoprotein cholesterol, *HDL-C* high density protein cholesterol, *Apo* apolipoprotein, *URIC* uric acid, *BUN* urea nitrogen, *Cr* creatinine.

### Gene expression changes in CSF patients

**Figure 2:**
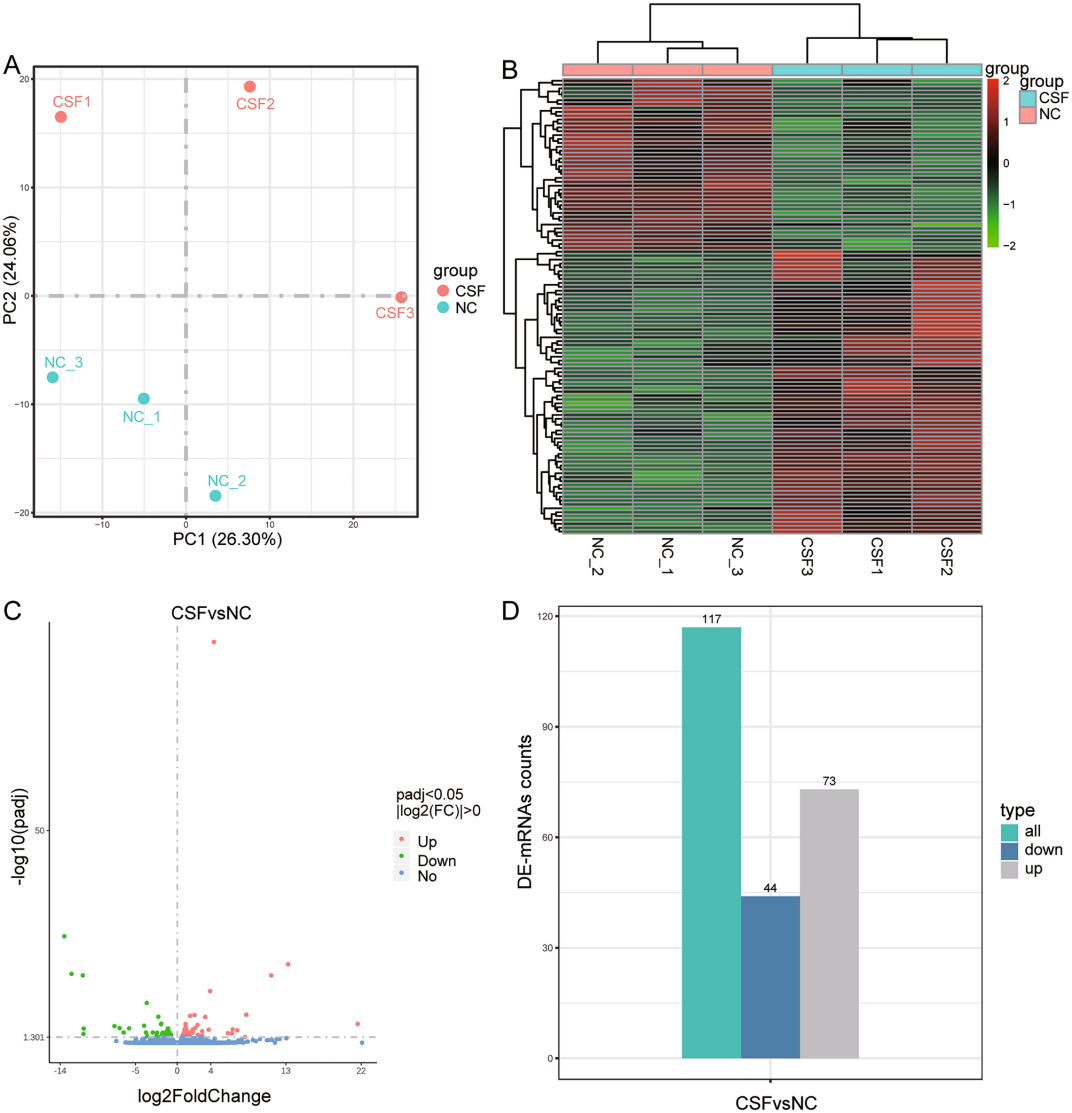
Identification of differentially expressed mRNAs between CSF and control. (**A**) The result of PCA for CSF and control samples. *CSF* coronary slow flow, *NC* healthy controls, *PC* principal component. (**B**) Heatmap of differentially expressed mRNAs in CSF and control samples. *CSF* coronary slow flow, *NC* healthy controls. Red is upregulated expression and green is downregulated expression. (**C**) Volcano map of differentially expressed mRNAs. Red is upregulated expression and green is downregulated expression. (**D**) Statistical histograms of up- or down-regulated mRNAs. Green is the number of all the differentially expressed mRNAs, gray is the upregulated expression and blue is the downregulated expression. *CSF* coronary slow flow, *NC* healthy controls.

First, we analyzed the degree of outlier between CSF and control samples. The principal component analysis (PCA) results showed that the similarity between the two groups of samples was low (Fig. 2A). By comparing the differentially expressed mRNAs (DE-mRNAs) between CSF and control, we obtained a significant differential expression of 117 genes, 96 of which were known (Fig. 2B,C, Table S1). There were 73 genes significantly up-regulated and 44 genes significantly down-regulated (Fig. 2D).

### Biological functions of differentially expressed mRNAs enrichment

**Figure 3:**
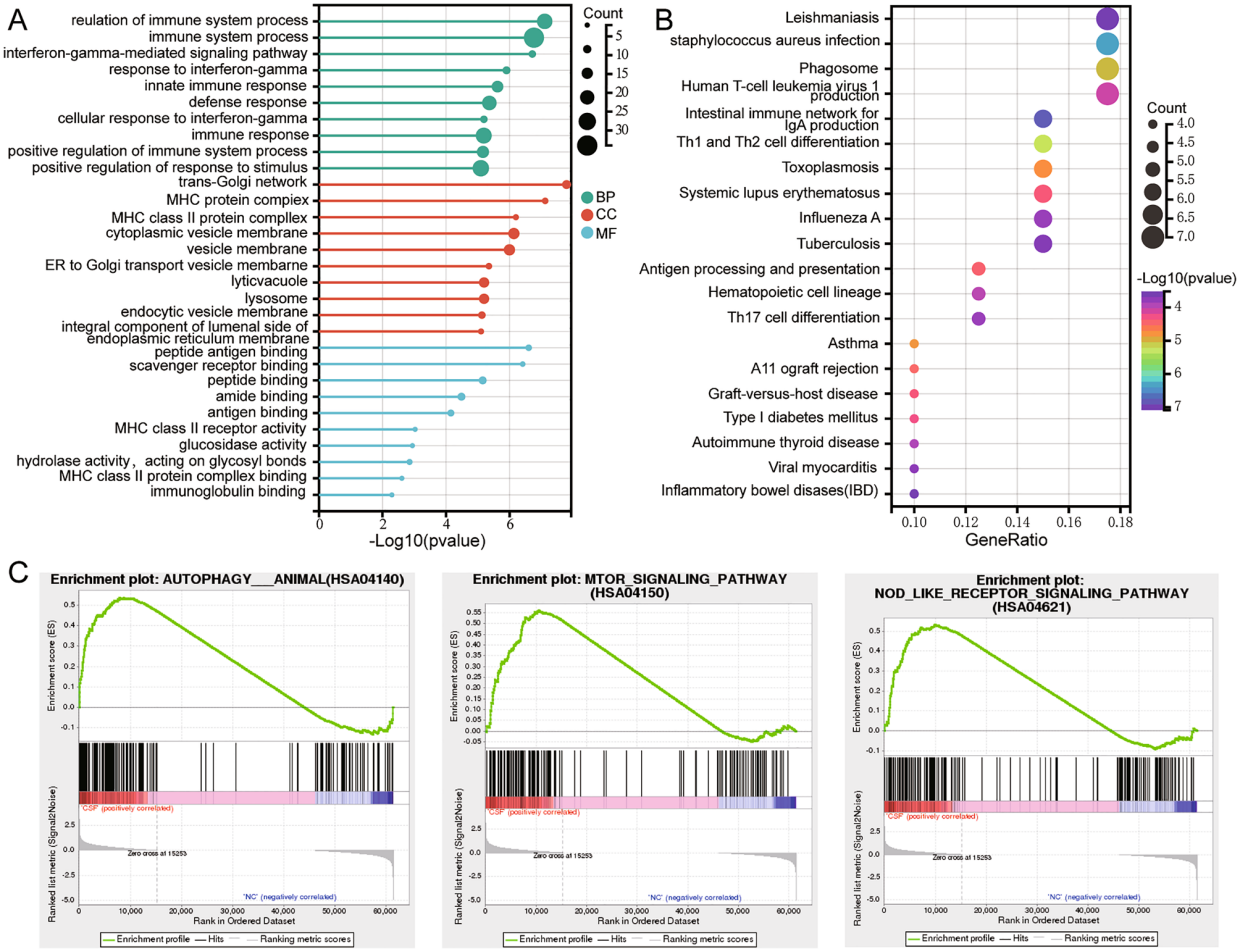
GO and KEGG terms for differentially expressed mRNAs. (**A**) The main biological processes (BP), cell composition (CC) and molecular function (MF) of differentially expressed mRNAs enrichment. (**B**) The KEGG pathway of differentially expressed mRNAs enrichment. The larger the dot, the more mRNAs involved in the pathway. The redder the color, the more significant. (**C**) The results of GSEA for mRNAs in CSF relative to controls. Adjusted *P* < 0.05 was considered statistically significant. *CSF* coronary slow flow, *NC* healthy controls.

In GO results (Fig. 3A), biological process (BP) mainly involved immune system process, interferon-gamma-mediated signaling pathway, and immune response. The cell composition (CC) mainly involved MHC class II protein complex, vesicle membrane, and lysosome. The molecular function (MF) mainly involved antigen binding, peptide binding and MHC class II protein complex binding. KEGG results showed that DE-mRNAs were mainly enriched in staphylococcus aureus infection, Th1 and Th2 cell differentiation, and Th17 cell differentiation (Fig. 3B). In addition, GSEA found that autophagy animal, mTOR signaling pathway, and NOD-like receptor signaling pathway were significantly enriched by CFS (Fig. 3C).

### Differentially expressed miRNAs in CSF

**Figure 4:**
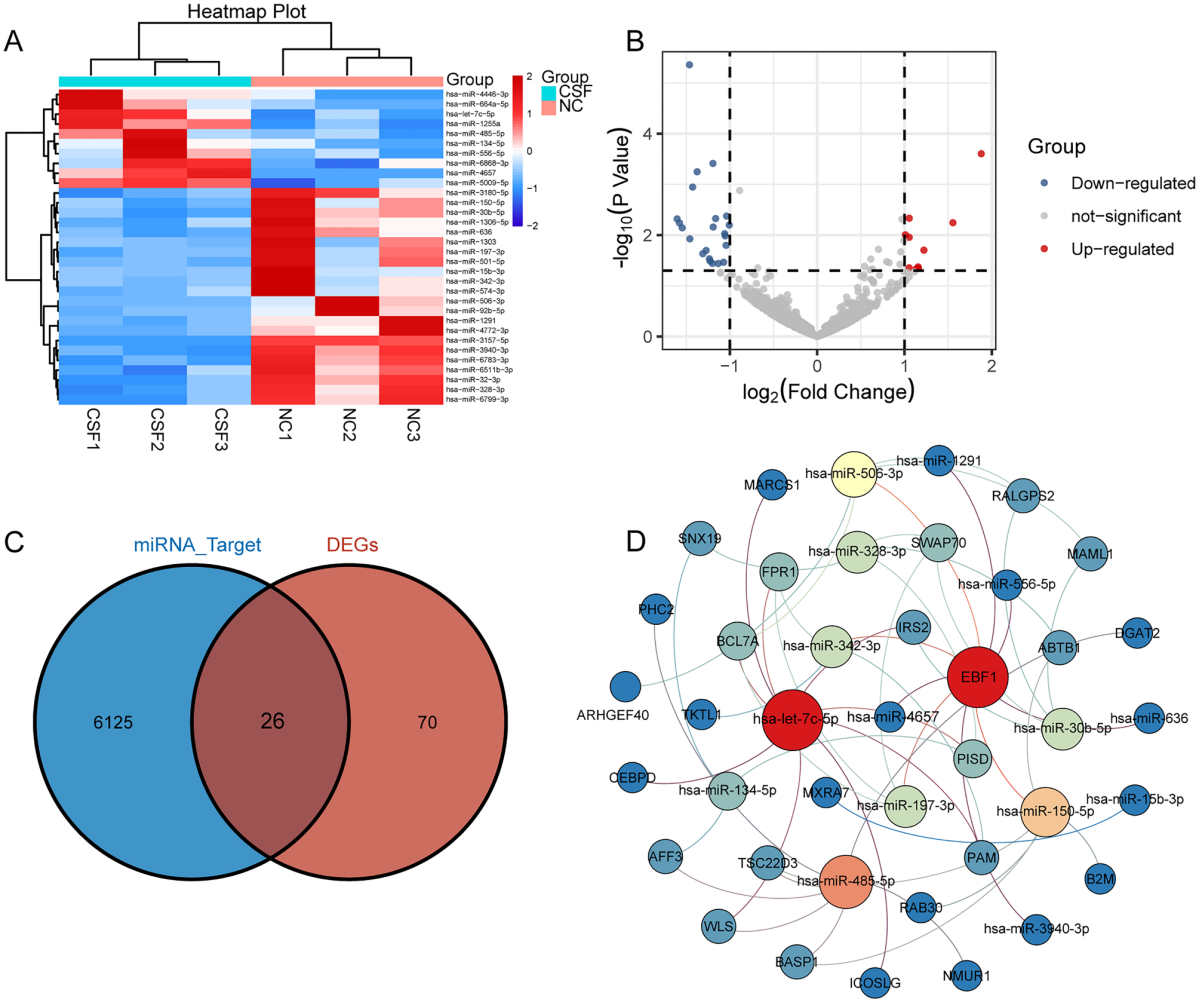
Differentially expressed miRNAs and regulatory networks. (**A**) Heatmap of differentially expressed miRNAs. Red represents significant upregulation in CSF; blue represents significant downregulation. *CSF* coronary slow flow, *NC* healthy controls. (**B**) Volcano map of differentially expressed miRNAs. Red represents significant upregulation in CSF; blue represents significant downregulation. (**C**) Intersection of target genes of differentially expressed miRNAs with differentially expressed mRNAs. (**D**) The miRNA regulatory network for intersects target genes. Circles are colored from blue to red, representing connected lines from less to more.

By comparing the differences between CSF and control, we obtained 32 differentially expressed miRNAs (DE-miRNAs) (Fig. 4A). These included 22 up-regulated and 10 down-regulated miRNAs (Fig. 4B). In the results of predicting, 6151 target genes of DE-miRNAs were obtained. By intersection analysis, we found 26 DE-mRNAs served as target genes (Fig. 4C). Then we constructed a regulatory network of 15 DE-miRNAs (Fig. 4D).

### Identification of key regulatory networks

**Figure 5:**
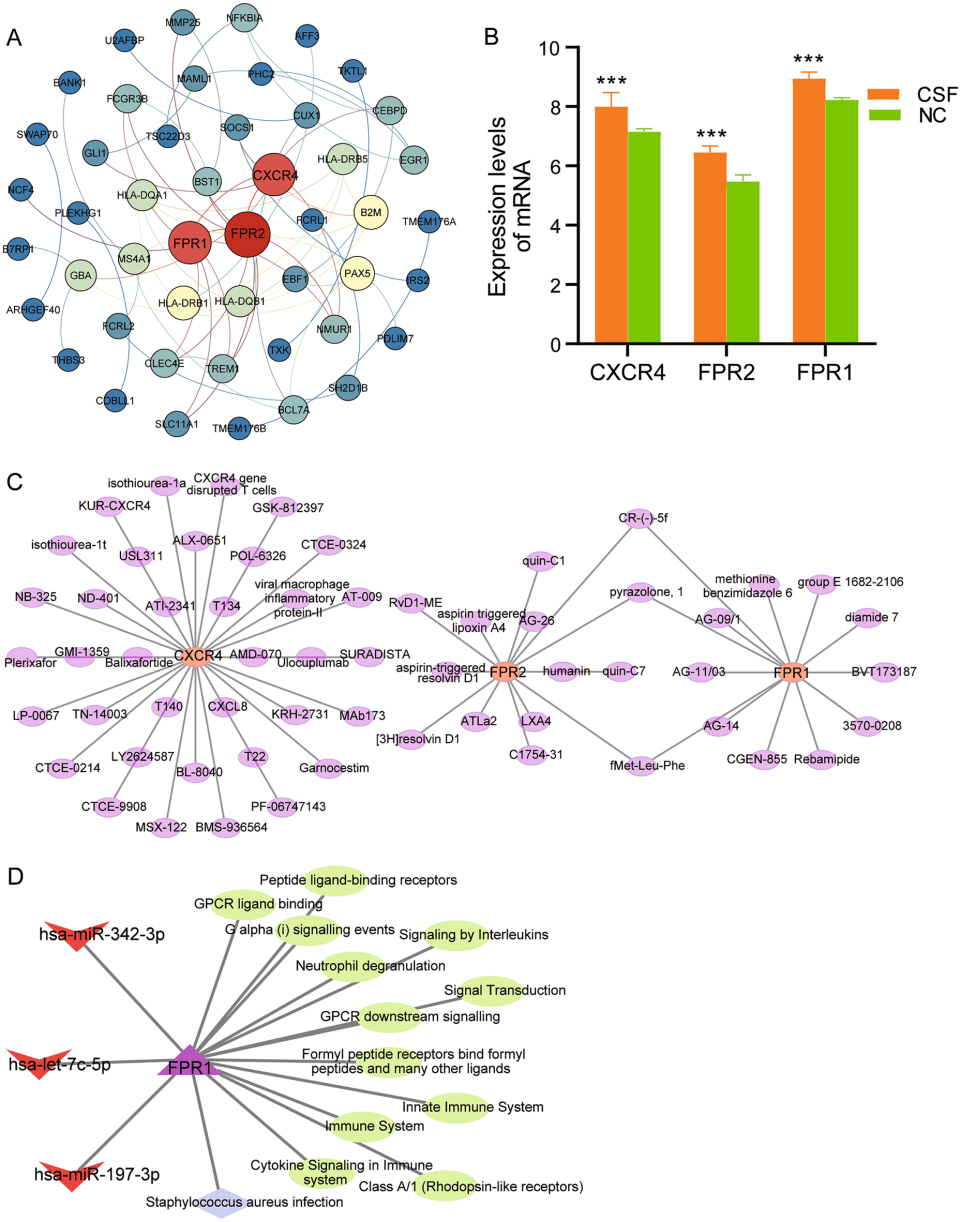
The miRNA regulatory network of hub genes. (**A**) PPI network of known differentially expressed mRNAs. Circles are colored from blue to red, representing higher connectivity of mRNAs in the network. (**B**) Differential expression levels of hub genes. ***P < 0.001. *CSF* coronary slow flow, *NC* healthy controls. (**C**) Predicted drugs targeted by hub genes in Therapeutic Target Database. (**D**) MiRNAs regulated hub gene to participate in biological functions. Triangle represents hub gene; diamond represents KEGG pathway; ellipse represents GO terms; V represents miRNAs.

In the analysis of the PPI network constructed from DE-mRNAs, we identified hub genes (FPR1, FPR2 and CXCR4) by degree (Fig. 5A). Compared with the control group, all three genes were upregulated expression in CSF (Fig. 5B). For drug prediction, we found 60 drugs targeted by hub genes (Fig. 5C). Among them, CR-(-)-5f, pyrazolone-1, and Met-Leu-Phe were targeted by both FPR1 and FPR2. In addition, FPR1 was the target gene of DE-miRNAs, including hsa-miR-342-3p, hsa-let-7c-5p and hsa-miR-197-3p (Fig. 5D). Hsa-miR-342-3p and hsa-miR-197-3p were down-regulated differences, while hsa-let-7c-5p were up-regulated differences. They regulated FPR1 to participate in immune inflammation-related biological functions.

**Figure 6:**
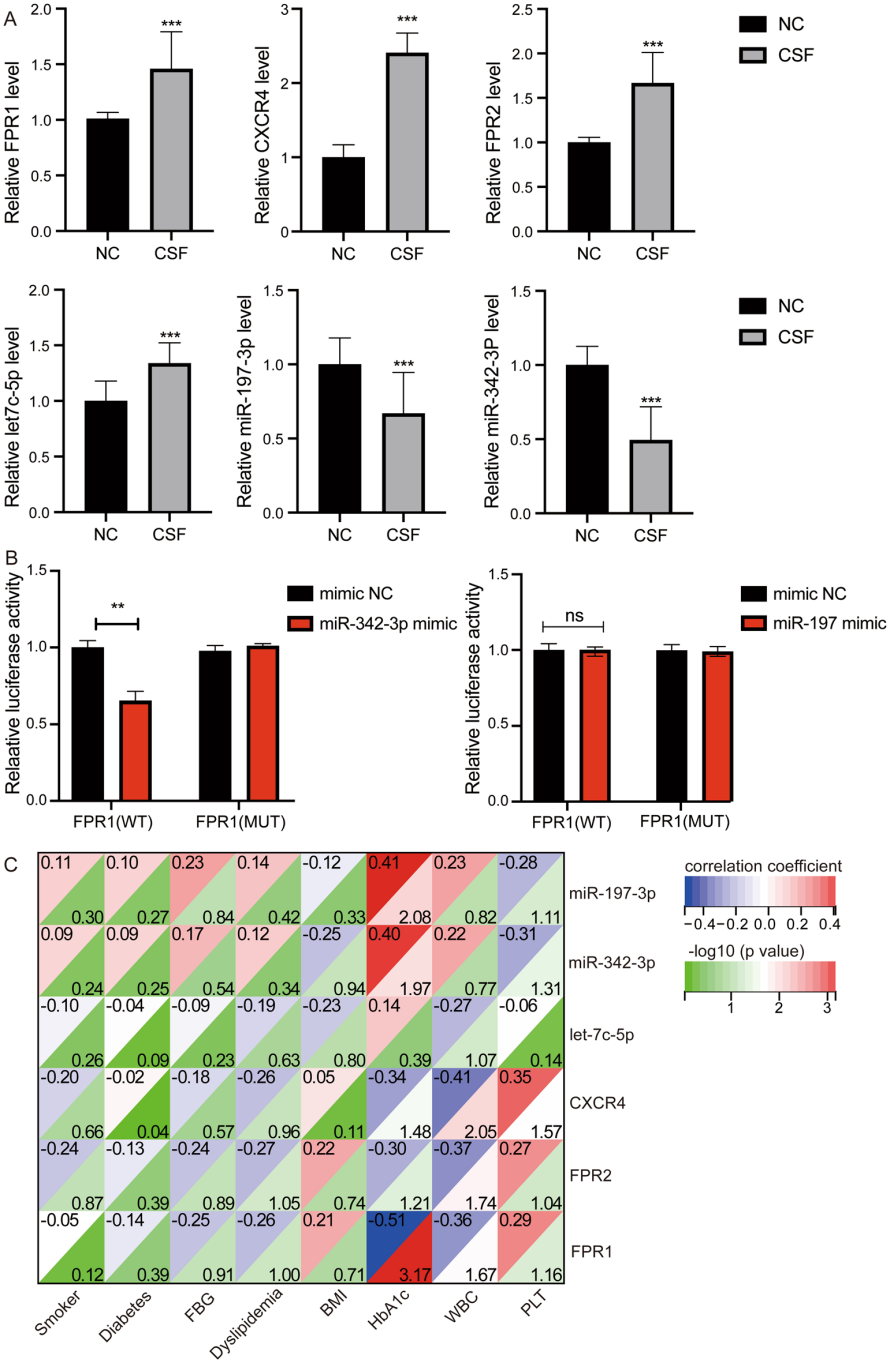
Molecular experimental validation. (**A**) Detection of differential expression of key miRNAs and hub genes between CSF and controls through qRT-PCR. ***P < 0.001. (**B**) Dual luciferase reporter system detects targeted binding between FPR1 and hsa-miR-342-3p or hsa-miR-197-3p. **P < 0.01. *CSF* coronary slow flow, *NC* healthy controls.

Importantly, through qRT-PCR experiments, we validated the differential expression of FPR1, FPR2 and CXCR4 between CSF and controls (Fig. 6A). Differences in key miRNAs were also validated. Then, we using dual luciferase reporter system to detect binding between FPR1 and hsa-miR-342-3p or and hsa-miR-197-3p. The results showed that luciferin activity decreased significantly in FPR1-WT and hsa-miR-342-3p mimic (Fig. 6B). In addition, the correlation among main clinical features and hub genes were shown in Fig. 6C. FPR1 was significantly positively correlated with body mass index (BMI) and platelet count (PLT), and negatively correlated with glycated hemoglobin (HbA1c) and white blood cell count in blood routine (WBC).

### MiR-342-3p inhibits cell proliferation

**Figure 7:**
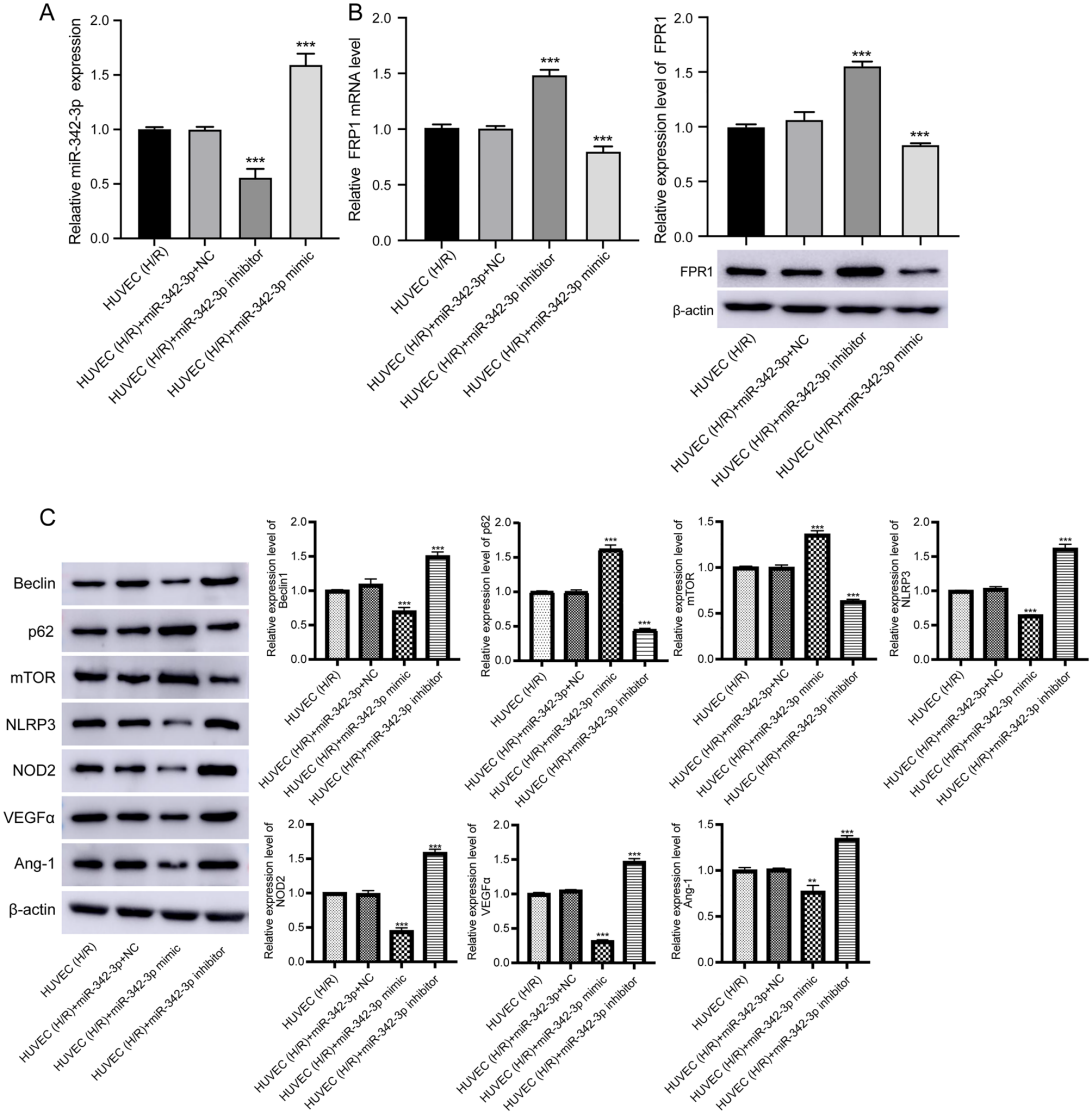
Expression of miR-342-3p, FPR1 and KEGG pathways in HUVEC. (**A**) The mRNA levels of miR-342-3p after HUVEC with H/R transfection with miR-342-3p mimics and miR-342-3p inhibitor. (**B**) The mRNA (left) and protein (right) levels of FPR1 after HUVEC with H/R transfection with miR-342-3p mimics and miR-342-3p inhibitor. ***P < 0.001. Original blots are presented in Supplementary Fig. 1A. (**C**) The protein levels of proteins of autophagy, mTOR signaling pathway, NOD-like receptor signaling pathway, and vasculogenesis. Original blots are presented in Supplementary Fig. 1B.

Firstly, the expression of miR-342-3p was significantly increased after transfection with miR-342-3p mimics, and was significantly reduced after transfection with the miR-342-3p inhibitor (Fig. 7A). Both qRT-PCR and Western blot results indicated that the expression of FPR1 significantly decreased after transfection with miR-342-3p mimics and increased significantly after transfection with the miR-342-3p inhibitor (Fig. 7B).

For KEGG pathways, after transfection with the miR-342-3p mimics, p62, and mTOR were increased, Beclin1, NLRP3, NOD2, VEGFA, and Ang-1 were decreased. This situation is opposite to transfection with the miR-342-3p inhibitor (Fig. 7C).

**Figure 8:**
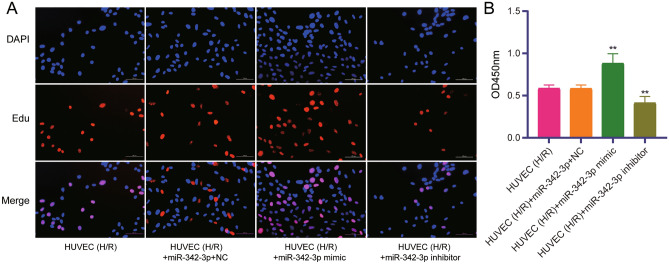
Detection of cell proliferation. (**A**) Proliferation of H/R treated HUVEC detected by Edu staining. (**B**) Proliferation of H/R treated HUVEC detected by CCK-8 method. **P < 0.01.

Moreover, the proliferation of HUVEC with H/R was increased after transfection with the miR-342-3p mimics, while cell proliferation inhibited after transfection with miR-342-3p inhibitor (Fig. 8A). Result of CCK8 showed that cell proliferation was increased in H/R HUVEC transfected with miR-342-3p mimics, and decreased in H/R HUVEC transfected with miR-342-3p inhibitor (Fig. 8B).

**Figure 9:**
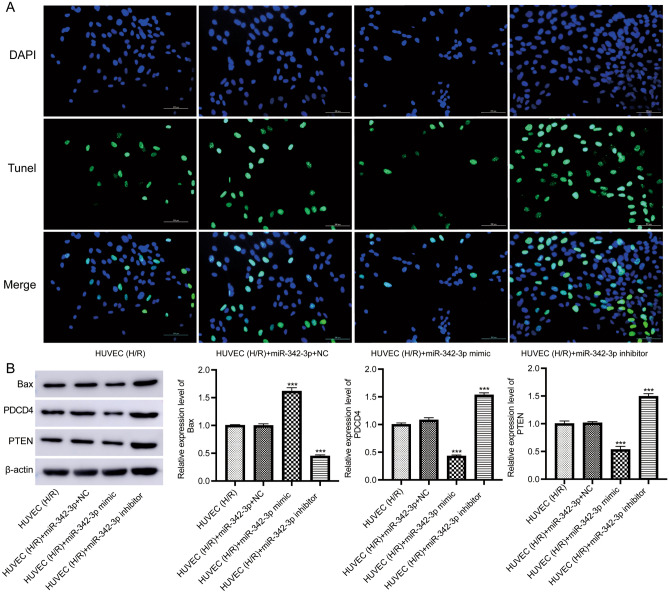
Detection of cell apoptosis. (**A**) The apoptosis of H/R treated HUVEC detected by TUNEL staining. Scale bar = 100 µm. (**B**) The protein levels of proteins related to apoptosis. Original blots are presented in Supplementary Fig. 2.

For cell apoptosis, it significantly decreased in the group with the miR-342-3p mimics, and significantly increased after transfection with miR-342-3p inhibitor (Fig. 9A). Western blot result showed that the expression of apoptotic proteins (Bax, PDCD4, and PTEN) was significantly reduced in the group with the miR-342-3p mimics and significantly increased in the group with the miR-342-3p inhibitor (Fig. 9B).

## Discussion

Although CSF has been known to cardiologists for about 40 years, its etiology and pathophysiological mechanisms are not well understood. This study aimed to explore the potential molecular mechanisms and biomarkers of CSF by high-throughput sequencing of CSF and control samples. Our analysis results showed that there were large differences in gene expression between CSF and control. This is not only closely related to the immune inflammatory response, but also regulated by miRNAs.

Among the biological functions enriched by the differentially expressed mRNAs, most were related to the immune inflammatory response. B cells produce antibodies that interfere with cardiomyocyte function as a result of the recruitment and activation of various innate and structural cell populations, including neutrophils, macrophages, fibroblasts and T cells^19^. T cells are a major component of the adaptive immune response. In the past decade, T cell immune responses have also played a central role in the pathophysiological pressure of human cardiovascular diseases^20^. Interferon-gamma may promote the development of CSF pathophysiology through endothelial dysfunction associated with inflammatory processes^21^. Inappropriate expression of MHC II genes may lead to a large number of inflammatory, infectious or autoimmune diseases^22^. The mTOR signaling pathway promotes cell growth and proliferation, including endothelial cells, by activating ribosome biogenesis^23^. These results suggest that dysregulated immune related biological effects may be a major pathological mechanism in CSF.

Through the PPI network, we identified three hub genes which were all high expressed in CSF. Although there is no direct evidence that hub genes were involved in the development of CSF, our analysis results suggested that they may play an important role in CSF. Early reports showed that formyl peptide receptor (FPR) played a role in regulating coronary and pulmonary artery tone^24,25^. The expression of FPR1 in activated macrophages and other leukocytes has been reported and is involved in the inflammatory response of endothelial cells^26,27^. Recent studies have shown that FPR1 can chemoattract leukocytes to the inflammatory site and drive the inflammatory response^28^. Activation of FPR2 can also induce the proinflammatory phenotype of endothelial cells^29^. FPR2 promotes the migration and proliferation of smooth muscle cells in atherosclerosis and mediates pro-inflammatory responses^30^. FPRs are expressed in T lymphocytes and may contribute to the promoting of inflammatory diseases^31,32^. FPR1 and FPR2 were both up-regulated expression in lymphocytes of the CSF and may contribute to the inflammatory response. The decreased proportion of lymphocytes may be a potential predictor of CSF^33,34^. T lymphocyte effectors are involved in the inflammatory response that leads to an increase in blood pressure^35^. Inflammatory phenomena associated with changes in platelet properties may also be related to CSF^36^. CXCR4 is involved in leukocyte chemotaxis under specific inflammatory conditions^37^. CXCR4 mediated progenitor cell mobilization has also been extensively studied in models of restenosis after vascular injury^38^. Local upregulation of CXCR4 in injured arterial wall may be one of the causes of intimal hyperplasia^39^.

In addition, on the drug prediction results, we identified potential therapeutic agents targeting the hub genes FPR1, FPR2, and CXCR4. The targeted drugs, such as CR-(−)-5f, pyrazolone-1, and Met-Leu-Phe, were predicted to potentially modulate these pathways, offering new avenues for the treatment of CSF. The identification of these genes could lead to the development of novel diagnostic tools that enable earlier and more accurate diagnosis of CSF, facilitating timely intervention.

MiRNAs can regulate protein expression at the post-transcriptional level, thus playing an important role in physiological and pathological processes. MiRNAs may serve as biomarkers for disease diagnosis and prognosis^40^. Compared with healthy controls, there is dysregulation of miRNAs in the blood of CSF patients^41^. Our analysis also showed that a large number of miRNAs were differentially expressed between CSF and control. And has a regulatory effect on differentially expressed mRNAs. FPR1 is regulated by hsa-miR-342-3p, hsa-let-7c-5p and hsa-miR-197-3p in the miRNA regulatory network of the CSF. MiR-342-3p, an obesity-related miRNA, has recently been recognized as a proangiogenic factor. Downregulated in endothelial cells of type 2 diabetes mellitus mouse models and human diabetic patients aggravates endothelial dysfunction by slowing proliferation and endothelial migration^42^. Let-7c-5p was significantly up-regulated in ascending thoracic aortic aneurysms^43^. Although let-7c-5p is also up-regulated in CSF in our results, the specific role is unclear. MIR-197-3p regulates the proliferation and migration of Kawasaki disease endothelial cells by targeting IGF1R and Bcl-2^44^. The expression of miR-197-3p was positively correlated with the expression of transforming growth factor beta 1, which was correlated with the nature of plasma clots^45^.

Subsequently, we further investigated the effect of miR-342-3p on FPR1 expression. Transfection with miR-342-3p mimics significantly reduced the expression of FPR1, further confirming that miR-342-3p can negatively regulate the expression of FPR1. Upregulation of miR-342-3p expression can promote the proliferation of HUVEC cells and promote cell apoptosis, suggesting that miR-342-3p may participate in the regulation of cell proliferation and apoptosis by regulating the expression of FPR1. This will offer new avenues for therapeutic intervention. Additionally, the positive correlation between FPR1 expression and BMI and PLT suggested that FPR1 might mediate some of the adverse cardiovascular effected associated with increased body weight, thrombogenesis or platelet activation. The negative correlation between FPR1 expression and HbA1c and WBC indicated the influence in immune and inflammatory response.

Our article also had certain limitations. First, the sample size of our sequencing experiments was small, although the sample size of later validation experiments increased. Secondly, we only validated the expression of key genes validated by qRT-PCR experiments, and the experimental means were relatively single. Then, whether the screened hub genes can be used as biomarkers and how was the diagnostic accuracy for CSF remains to be further studied.

## Conclusion

This study identified CSF-related molecular mechanisms and potential biomarkers by high-throughput sequencing. The results suggested that immune inflammation played an important role in the disease process of CSF. We identified FPR1, FPR2 and CXCR4 as potential biomarkers of CSF. The hsa-miR-342-3p, hsa-let-7c-5p and hsa-miR-197-3p were key regulators. Ultimately, we identified that FPR1 was regulated by key miRNAs and participates in the CSF network through immune inflammatory response. In conclusion, our study offers valuable insights into the molecular regulation mechanisms and potential biomarkers of CSF, with significant implications for the early diagnosis, prognosis, and personalized treatment of this condition.

### Supplementary Information

Supplementary Figure S1.

Supplementary Figure S2.

Supplementary Table S1.

## Data Availability

The datasets used and/or analysed during the current study available from the corresponding author on reasonable request.
