# Supplementary material for: Differential gene expression and miRNA regulatory network in coronary slow flow
Source: Sci Rep. 2024 Apr 10;14:8419. doi: 10.1038/s41598-024-58745-w (PMC11006858; doi:10.1038/s41598-024-58745-w)
Supplement: Supplementary file 1 — Supplementary Figure S1. [file 41598_2024_58745_MOESM1_ESM.pdf]

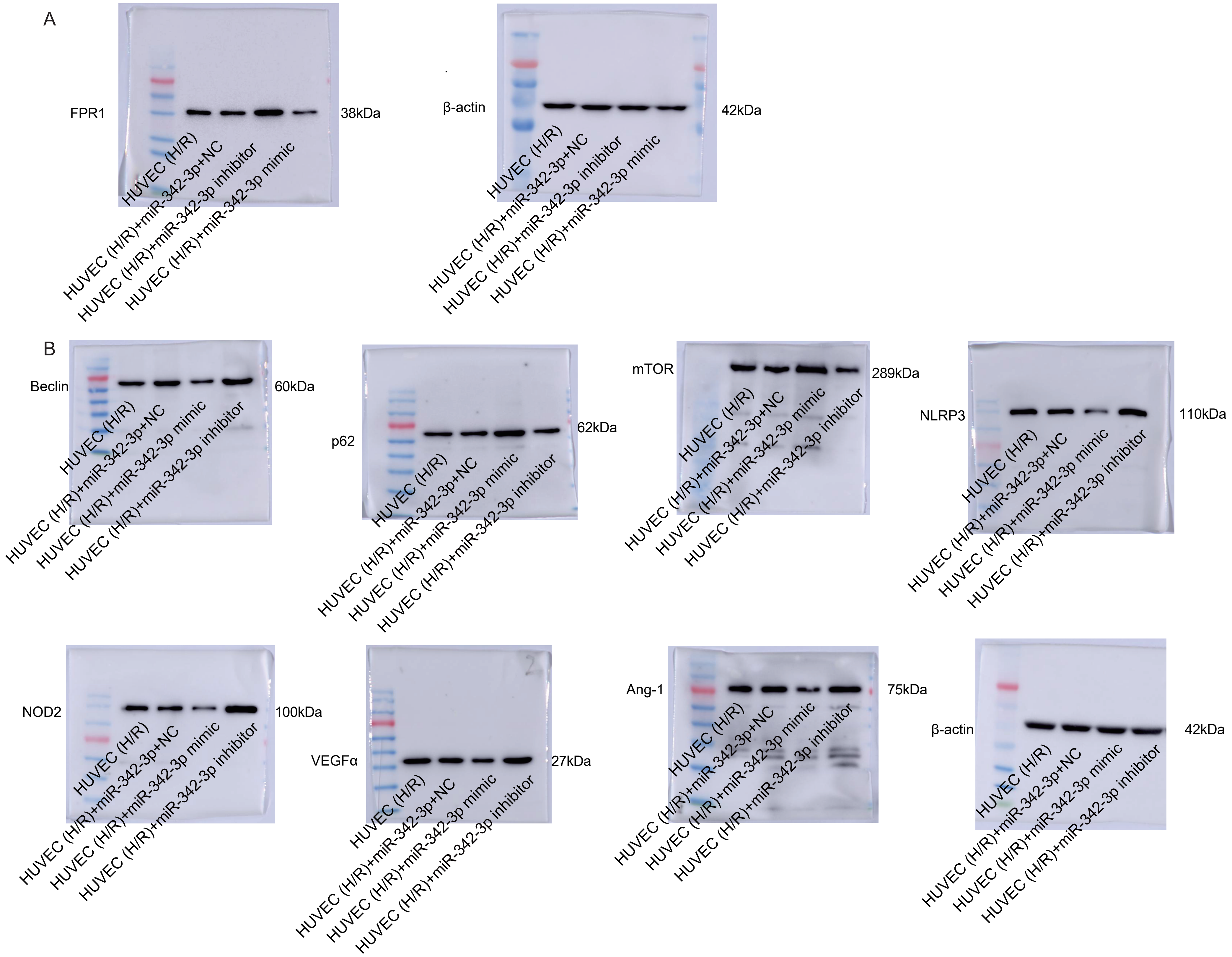


Figure S1. Images of expression of FPR1 and proteins in KEGG pathways after HUVEC treated with H/R transfection with miR-342-3p mimics and miR-342-3p inhibitor detected by Western blot.
