# Supplementary material for: Differential gene expression and miRNA regulatory network in coronary slow flow
Source: Sci Rep. 2024 Apr 10;14:8419. doi: 10.1038/s41598-024-58745-w (PMC11006858; doi:10.1038/s41598-024-58745-w)
Supplement: Supplementary file 2 — Supplementary Figure S2. [file 41598_2024_58745_MOESM2_ESM.pdf]

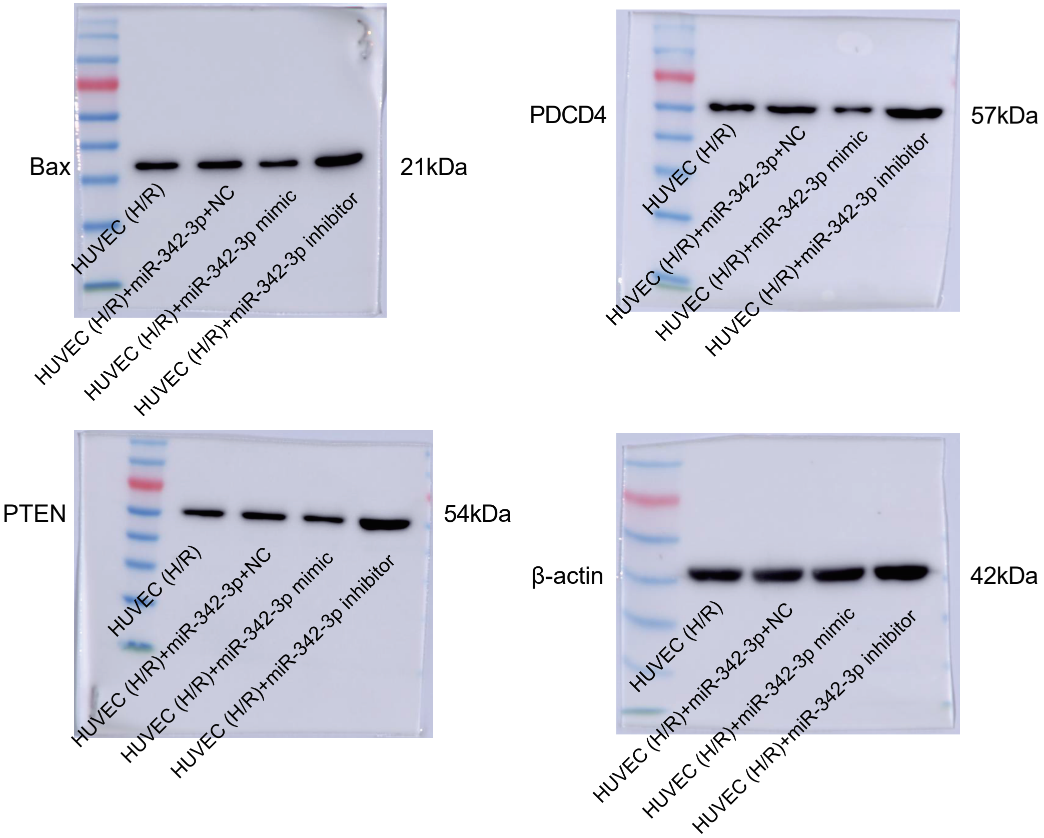

Figure S2. Images of expression of apoptosis proteins after HUVEC treated with H/R transfection with miR-342-3p mimics and miR-342-3p inhibitor detected by Western blot.
